# After the lockdown: simulating mobility, public health and economic recovery scenarios

**DOI:** 10.1038/s41598-020-73949-6

**Published:** 2020-10-12

**Authors:** Alessandro Spelta, Andrea Flori, Francesco Pierri, Giovanni Bonaccorsi, Fabio Pammolli

**Affiliations:** 1 Department of Economics and Management, University of Pavia, Via San Felice 7, 27100 Pavia, Italy; 2 Impact, Department of Management, Economics and Industrial Engineering, Politecnico di Milano, Via Lambruschini, 4/B, 20156 Milan, Italy; 3 Department of Electronics, Information and Bioengineering, Politecnico di Milano, Via Giuseppe Ponzio 34/5, 20133 Milan, Italy; 4 CADS, Joint Center for Analysis, Decisions and Society, Human Technopole, Via Cristina Belgioioso, 171, 20157 Milan, Italy

**Keywords:** Mathematics and computing, Diseases

## Abstract

The spread of SARS-COV-2 has affected many economic and social systems. This paper aims at estimating the impact on regional productive systems in Italy of the interplay between the epidemic and the mobility restriction measures put in place to contain the contagion. We focus then on the economic consequences of alternative lockdown lifting schemes. We leverage a massive dataset of human mobility which describes daily movements of over four million individuals in Italy and we model the epidemic spreading through a metapopulation SIR model, which provides the fraction of infected individuals in each Italian district. To quantify economic backslashes this information is combined with socio-economic data. We then carry out a scenario analysis to model the transition to a post-lockdown phase and analyze the economic outcomes derived from the interplay between (a) the timing and intensity of the release of mobility restrictions and (b) the corresponding scenarios on the severity of virus transmission rates. Using a simple model for the spreading disease and parsimonious assumptions on the relationship between the infection and the associated economic backlashes, we show how different policy schemes tend to induce heterogeneous distributions of losses at the regional level depending on mobility restrictions. Our work shed lights on how recovery policies need to balance the interplay between mobility flows of disposable workers and the diffusion of contagion.

## Introduction

SARS-COV-2 has caused almost 7 million confirmed cases and 400k fatalities globally as of June 6th 2020^1^. Consequences on economic activities and trade flows have been strong^2–4^. In addition to the direct effects of the contagion, mobility restriction policies have been put in place in many countries to limit the spread of SARS-COV-2. Indeed, during pandemics, different types of non-pharmaceutical interventions (NPIs) have been implemented to contain the spread of contagion^5^. These policy restrictions have produced relevant economic consequences, since disposable workers (i.e., individuals not infected and available to work) are prevented from keeping up their activities^6–9^.

This work aims to study the interplay between the SARS-COV-2 diffusion and mobility restriction measures, and how it affects the Italian productive system. Through a scenario analysis which models the transition into a post-lockdown phase, we investigate the economic consequences of alternative lockdown lifting policies. We study how economic outcomes are influenced by (a) the severity of virus transmission rates across Italian regions and (b) the timing and intensity of the release of mobility restrictions^10,11^. Italy has been the first European country to experience a severe SARS-COV-2 pandemic^12^, with very high mortality rates in some of the northern regions. Moreover, it has been the first country to impose strong lockdown measures on a national scale, despite an already weak prospected economic outlook: before SARS-COV-2 outbreak, Italy was in fact experiencing some of the lowest growth rates in Europe (according to Eurostat), while the first quarter of 2020 indicates a fall of Gross Domestic Product (GDP) of about 5.3% with respect to previous quarter (according to the Italian Institute of Statistics). Against this background, Italy represents an interesting case study to simulate lockdown lifting scenarios, due to a peculiar trade-off between economic and health conditions.

In this work the spreading of the virus is modeled through a metapopulation SIR model^13–20^ that simulates SARS-COV-2 diffusion in Italy. We partition the Italian population into districts^21^ and we introduce commuters that, moving between different districts, contribute to spread the disease (see “Methods”).

Despite epidemic models identify specific mechanisms relevant to characterize virus diffusion and the social implications of the epidemic^22^, policymakers barely handle the discrepancies between different models^23^. To cope with this issue we introduce a model-based scenario analysis for SARS-COV-2 calibrated on real mobility flows data. We leverage a massive dataset of aggregated and de-identified information on mobility flows between Italian municipalities, provided by Facebook through its “Data for Good” program^24^. Data account for daily movements of approximately 4 M individuals in a period of 1 month, from February 24th to March 24th. In line with Refs.^11,25^, we simulate the course of the epidemic by calibrating our simulations with parameters obtained from recent studies on SARS-COV-2^26,27^.

Our analysis relates with contemporary literature about the side effects on the economic consequences of measures put in place to circumvent the pandemic. Similar to Ref.^28^, we perform a scenario analysis to investigate a set of lockdown lifting schemes (moving to the so-called “Phase-2”), to highlight the challenge for policymakers in reaching a proper balance between the spread of contagion and economic recovery^29,30^. To investigate this trade-off, we explore the range of economic outcomes deriving from different lockdown lifting schemes for the Phase-2. We imagine two extreme cases: one, in which mobility restrictions continue while transmission rates come back to pre-lockdown values, the other, in which mobility is fully restored to pre-lockdown values while transmission rates are kept to the minimum reached during the lockdown phase. Then, we perform simulations with values in-between these two extreme cases by tuning parameters related to the transmission rate and to mobility flows.

To show how the complexity of Italian productive structure can magnify losses beyond the direct effects of SARS-COV-2, we measure the share of employed individuals at the sector level, in each Italian district. We collect socio-economic variables at the district level from the 15th General Census of Population and Housing developed by the Italian National Institute of Statistics (ISTAT). The database contains information, at sub-municipal levels, on the demographic and social structure of the Italian population. In particular for each district, we rely on information on population size, average income, and the number of workers in different economic sectors, available in the Employment Register created in 2011 on the occasion of the CIS2011 Virtual Business Census and updated annually, starting from 2012^31^. This information is matched with the details on the economic sectors which, starting from 22th March, were allowed to continue their activity during the lockdown phase^32^.

Thus, our estimates of the economic backslashes of the epidemic will result from the interplay of three sources of variability: the range of possible values of the epidemic parameters, the evolution of mobility patterns and the productive structure of the economy.

The role of mobility patterns in explaining contagion dynamics has been a central finding in the recent literature on SARS-COV-2^19,33–36^. Accordingly, public health interventions have focused mainly on mobility restrictions, with a varying degree of intensity: school closures, bans of public events, curfews, social distancing and self-isolation^37^. Given the rapid spread of contagion and the high death toll in several countries, lockdowns have been enforced to err on the side of caution, hence the effectiveness of each measure has not been accurately tested and the debate is still open on which intervention (or combination of interventions) may lead to successful results^38^. Nevertheless, increasing evidence has focused on highlighting the role of mobility restriction in effectively curbing the rate of contagion^25,29,39–43^, hence in what follows we will use a simplifying assumption and we will attribute the variation in the effective rate of reproduction during lockdown entirely to the variation in mobility. This allows us to focus only on those details which are functional to estimate the economic losses during lockdown and after lockdown lifting. For the same reason, in the scenario analysis for Phase-2, we do not model the epidemic dynamics and we explore the space of mobility and contagion parameters, using as starting point estimates of the effective reproduction number obtained from Ref.^27^.

While there is a well-established literature incorporating mobility patterns in epidemic modeling, economic models of epidemics have mostly focused on what is called the SIR macro model^44^, which assumes individuals uniformly distributed in space and randomly moving (see Ref.^45^ for an interesting modeling exception). On this issue, our work improves over previous models by using real data on movements of individuals in the simulations within a metapopulation model tailored to address the role of mobility in the contagion. On the other hand, however, we have used a simplified model of losses, which includes only direct losses from infections and deaths (and their health related costs) and job losses from NPIs. Following the classification made in Ref.^44^, there are three sources of economic damages which our model does not include: change in consumption and investing behavior of households and firms, disruption of trade and global value chains, and hysteresis effects, i.e. damages to the capacity of the global economy to achieve growth in the long run. Including all these effect in our model would increase our estimate of reported losses, but at the same time would also introduce new sources of uncertainty in the estimation procedure due to the number of parameters required to calibrate the model. For this reason we have chosen to focus here on the first two types of damages and use a more data-driven approach.

## A model for economic losses

The epidemic model that we adopt (see “Methods”) provides information about the spreading of SARS-COV-2 in Italy and, ultimately, on $I_{i}(t)$, the number of infected individuals in each district *i*. According to the model, restrictive policies which affect national mobility have a two-fold effect: first, they contribute to slow down the infection dynamics, thus increasing the stock of *disposable* workers (not infected); second, they also limit the number of *actual* workers which are allowed to produce. For the Italian case, indeed, during the lockdown phase only a subset of essential sectors was allowed to continue their economic activities, while the others were forced to close when remote work was not feasible^46–49^. We assume that ${\tilde{L}}_{i}^{\nu }(t)$, the *disposable* labor force (from now on we will use “labor force” and “workers” interchangeably) in the $\nu$th economic sector inside the *i*th district at each instant *t*, is a fraction of the *total* labor force ${\hat{L}}_{i}^{\nu }$, which depends on the ratio of infected people in that district with the respect to the total number of individuals in the population, *N*(*t*):

1 $$\begin{aligned} {\tilde{L}}^{\nu }_{i}(t)= \left( 1-\frac{I_{i}(t)}{N_{i}(t)} \right) \cdot {\hat{L}}_{i}^{\nu }. \end{aligned}$$

The *actual* labor force, i.e., the number of workers that are effectively active, on the contrary varies based on mobility restriction measures: it coincides with the disposable force during the pre-lockdown phase only (*Pre*), while during the lockdown phase (*Lock*) it is composed by the workers employed in the essential economic sectors which were allowed to continue their activities^50^ and, moreover, by the fraction of the disposable labor force which could perform “smart-working”, $\xi _{i}$^10^. Finally, during the Phase-2 (*Unlock*), we assume that a varying fraction $\delta _{1}$ of the available workers not working remotely are allowed to return to their job. Thus, we compute the actual labor force in district *i* as:

2 $$\begin{aligned} L_{i}(t)={\left\{ \begin{array}{ll} {\sum }_{\nu } {\tilde{L}}_{i}^{\nu }(t) &{} \text{ if } t \in Pre \\ {\sum }_{\nu \in E} {\tilde{L}}_{i}^{\nu }(t) + \xi _{i} {\sum }_{\nu \notin E} {\tilde{L}}_{i}^{\nu }(t)&{} \text{ if } t \in Lock\\ \delta _{1}(1-\xi _{i}) {\sum }_{\nu } {\tilde{L}}_{i}^{\nu }(t) +\xi _{i} {\sum }_{\nu } {\tilde{L}}_{i}^{\nu }(t) &{} \text{ if } t \in Unlock \end{array}\right. }, \end{aligned}$$

where *E* indicates the set of essential sectors allowed to produce during the lockdown phase. Notice that the parameter $\delta _{1}$ allows us to model different scenarios by linking together the evolution of mobility and the actual labor force: in our analysis we will explore various range of values for $\delta _1$ to simulate different degrees of openness in the lockdown lifting phase, assuming that the share of remote workers $\xi _{i}$ will instead remain constant.

To measure the aggregate economic loss for each district, we average the municipal level per-capita incomes to their corresponding district level, from which we obtain the daily district income per person ($w_{i}$). Moreover, we set the per-capita daily standard hospitalization cost at $c^{Hosp}=500$ euros^51^ and the daily Intensive Care Unit (ICU) cost at $c^{Icu}=1500$^52–54^. We then collect information on the fraction of infected individuals needing different kinds of medical care from the Italian Civil Protection bulletins^55^; in particular, we take the average of both quantities for each district over the entire period of observation to avoid fluctuations in the measurements. These measures must be seen as a lower-bound for costs since they refer to services provided during ordinary time and do not account for the extra costs deriving from the epidemic. Moreover, we do not attempt to make any estimate of costs incorporating the value of statistical life^56,57^. On the one hand our focus is to estimate economic losses only and not perform a cost-benefit analysis as in Refs.^58–60^. On the other hand, we want to be parsimonious about our set of assumptions.

The total cost to the healthcare system per district is thus found as:

3 $$\begin{aligned} C_{i}(t)=c^{Hosp} \cdot p^{Hosp}_{i} \cdot I_{i}(t) + c^{Icu} \cdot p^{Icu}_{i} \cdot I_{i}(t). \end{aligned}$$

The economic loss suffered by each district then is given by the sum of hospitalization costs and the loss of potential income, i.e., the difference between the potential income that could have been obtained in an epidemic-free scenario ($w_{i} \cdot {\hat{L}}_{i}(t)$) and the income resulting from the combined effect of mobility reduction and epidemic spreading. In formula:

4 $$\begin{aligned} \Delta _{i}(t) = \left[ w_{i} \cdot {\hat{L}}_{i}(t) - w_{i} \cdot L_{i}(t) \right] +C_{i}(t). \end{aligned}$$

In the experimental phase, we perform simulations assuming that the infection started in Northern Italy on 26th of January^61^. First confirmed cases of SARS-COV-2 were observed in Veneto and Lombardy between the 20th and 23rd of February, and we accordingly initialize our simulation assuming 100 infected people proportionally distributed among these regions^62^. Therefore, we set the duration of the pre-lockdown phase (*Pre*) to 42 days, from the 26th of January to 8th of March. We then impose that the lockdown phase (*Lock*) is put in place for 46 days, from the 9th of March up to the 3rd of May. Finally, we start Phase-2 (*Unlock*) on May 4th ($t=100$). The total simulations length ${{\mathscr {T}}}$ is set to 700 days, following recent estimates on the total duration of the epidemic^63,64^. In each phase we update accordingly the configuration of parameters (see “Methods” for details).

## Results

### Mobility reduction during the lockdown

To characterize the impact of NPIs we analyze the network of mobility at the level of municipalities (see “Methods”). In particular, we investigate with a network science approach the number of weakly connected components on a daily basis, and the size of the largest weakly connected component over a symmetric window of 2 weeks before/after the day of intervention (9th March). We report that the number of components increases from 394 on February 24th to 1216 on March 23rd, whereas the largest component during 2 weeks of national lockdown shrinks by only 16% in size (from 2733 to 2293 nodes).

**Figure 1:**
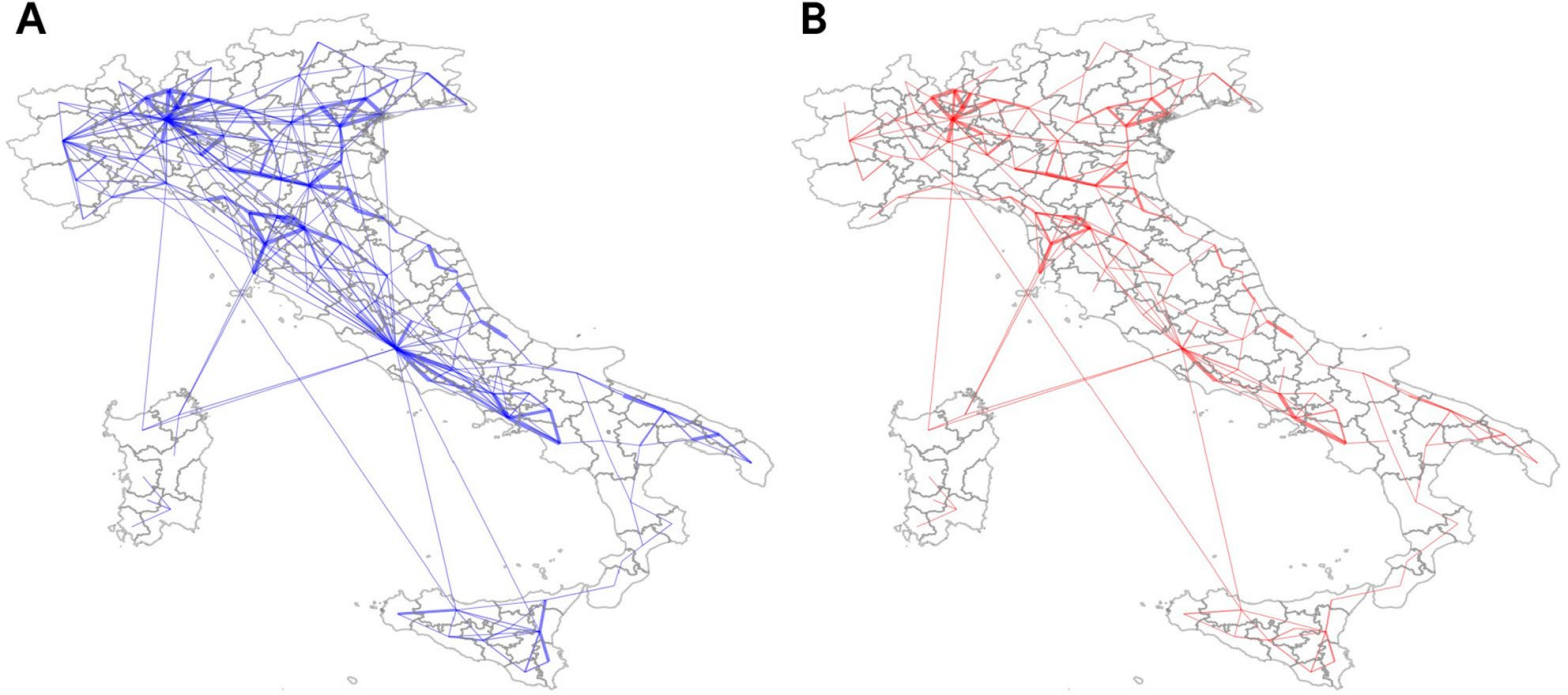
Mobility patterns in Italy before (**A**) and during (**B**) the lockdown. The figure shows the reduction of the national mobility induced by the lockdown measures of March 9th. We represent matrices of mobility at the district level as described in Eq. (6), i.e. panel (**A**) corresponds to ${\tilde{l}}^{Pre}$ and panel (**B**) to ${\tilde{l}}^{Lock}$. The thickness of edges is proportional to the inter-district mobility.

In Fig. 1, we show an illustrative example of the evolution of the Italian network of mobility at the district level before and during national closure. The main mobility hubs (Turin, Milan, Bologna, Rome, Naples) are still connected during lockdown, and the national transportation infrastructure is not disrupted. However, we do observe an important change in both volumes and distances traveled by individuals moving. In fact, long range connections almost disappear, and short distance trips increase (see Supplementary Fig. S1. These findings confirm the results of similar analyses built upon the same dataset provided by Facebook^31,65^: the Italian peninsula exhibits a distributed network of transportation which was severely affected by national closure but did not collapse.

To investigate the representativity of Facebook mobility data, we perform specific comparison exercises with the 2011 commuting network provided by the Italian National Institute of Statistics (ISTAT), which provides movements of workers/students travelling between municipalities that were recorded in the last census. We tread carefully in the comparison, first because the commuting network retains only residents who travel for either work or study reasons, and second because the information refers to the Italian mobility patterns of 9 years ago. On the other side this data is not biased towards individuals who own a phone and are registered on social networks, as in Facebook data, therefore we believe it is useful to further validate the representativity of the Italian mobility data observed before entering the lockdown phase. To make a consistent comparison we filtered out those paths where the number of travelers is less than 10 (in fact, Facebook uses this value as threshold to include an observation^24^) and retained only municipalities which are present also in our dataset (approx. 1/3 of all Italian municipalities). Besides, we build an averaged graph of mobility over a window of 14 days before national lockdown (9th March). Then, we performed Pearson’s correlation test to the following metrics: Degree and Strength of nodes, and Weight of edges. In all cases we find a significant positive correlation at level $\alpha =0.001$ (see Supplementary Fig. S2). These findings provide us with a solid background for our metapopulation model, as commuting networks are often employed together with airline traffic to model epidemics spreading^16^, designed at district level in order to match economic variables (see “Methods”).

### Epidemic dynamics

In this section we describe how national lockdown affected the evolution of the virus spreading in Italy in time and space. To this aim, we perform a comparative static exercise, in which we assume two basic scenarios (see “Methods” for details on the model and parameters). In the first scenario no mobility restrictions are applied, and all parameters remain at their *Pre*-lockdown values for the whole simulation length. In the second (more realistic) scenario national lockdown is first put in place and then lifted, assuming that the transmission rate is held constant (to its lockdown value) during the re-opening phase; however, as we show in the following, we mainly focus on the epidemic dynamics during the lockdown phase to assess the efficiency of restriction measures to contain the epidemic spreading. Hence from $t=0$ to $t=42$ parameters assume their *Pre*-lockdown values. During the lockdown phase (from $t=43$ to $t=99$) we set them to their *Lock* values. Finally, from May 4th (i.e., $t=100$ to $t=700$) we set $\beta ^{Unlock}=\beta ^{Lock}$, $l^{Unlock}=l^{Pre}$ and $r^{Unlock}=r^{Pre}$. We are aware that this second scenario is an over-optimistic case, but we remark that a precise forecast of the infection dynamics after lifting mobility restrictions is beyond the scope of this analysis. We limit to show that during lockdown phase our model is able to replicates some stylized facts observed during the course of the epidemic in Italy both temporally and geographically^20^. Therefore, we further rely on the model to evaluate the economic impact of the epidemic assuming a grid of values for both transmission rate and mobility flows in the post-lockdown phase.

**Figure 2:**
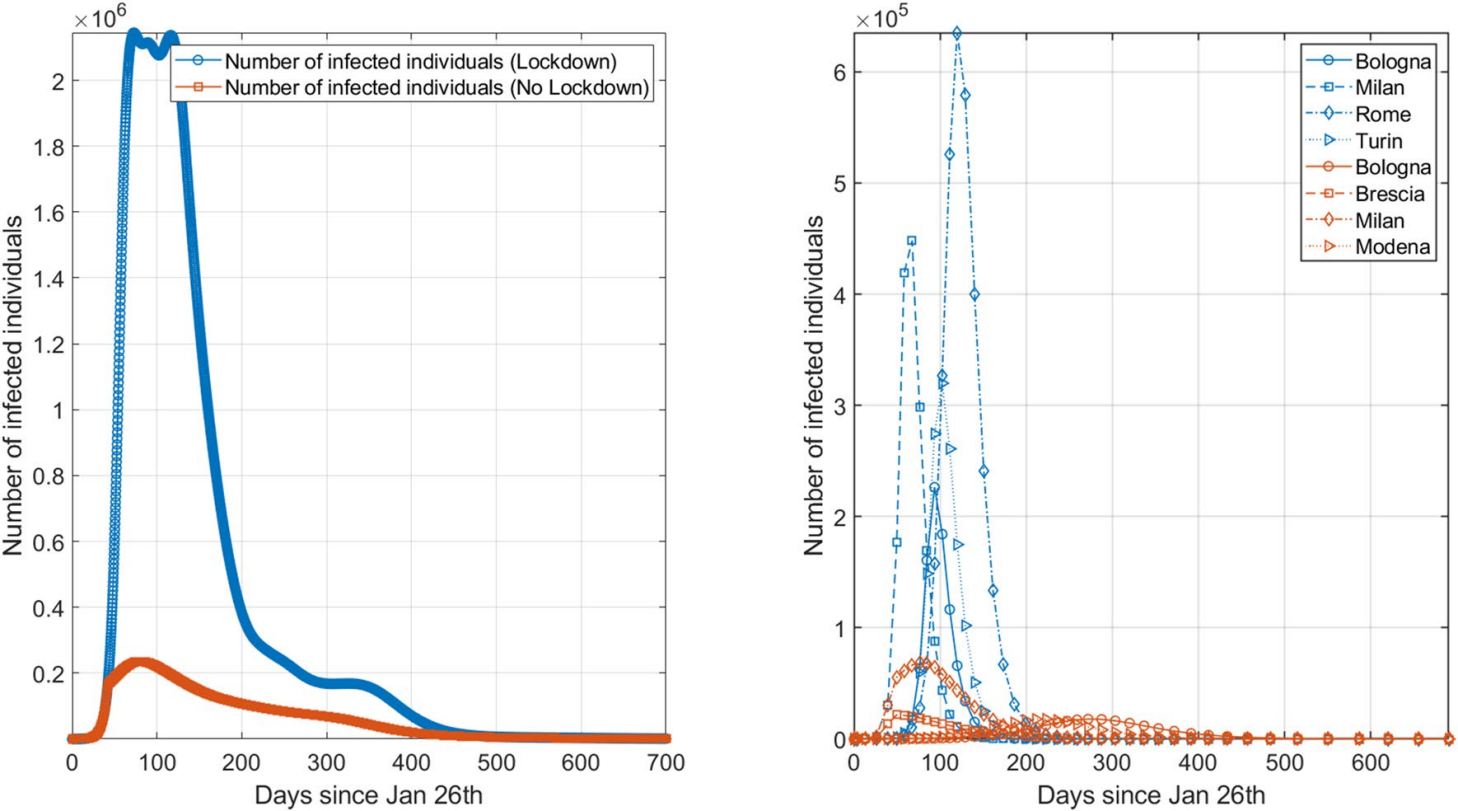
Evolution of the epidemic in Italy and most affected cities with and without intervention. Left panel represents the the dynamic of the total number of infected individuals in Italy in presence (orange line) or absence (blue line) of lockdown measures. Right panel shows the same information for the four most infected districts in the two scenarios.

Figure 2 (see also Supplementary Figs. S4–S5) shows the temporal dynamics of the virus spreading in the two aforementioned scenarios. In particular, left panel shows the evolution of the total number of infected individuals for the entire peninsula, with a focus on the four most affected districts in each scenario provided in the right panel. Orange lines correspond to the lockdown scenario case whereas blue lines stand for the absence of mobility restriction measures.

According to the model, we show that mobility restrictions successfully reduce the total number of infected individuals in Italy by one order of magnitude compared to the opposite scenario. Moreover, the curve of the infected individuals exhibits a peak around mid-April, in accordance with real measurements^62^. Districts in Northern Italy are the most infected areas in both scenarios, with only a limited spread of the virus in the rest of the peninsula in case of national closure (see Supplementary Figs. S4–S5), in accordance with the real data. In absence of mobility restriction policies, on the other hand, the epidemic spreads along the high-speed rail line from Lombardy down to south (e.g., Florence, Rome, Naples), in line with Ref.^36^. Instead, when mobility restrictions are effectively put in place the virus reaches Emilia-Romagna districts (i.e., Modena and Bologna), whereas southern regions are spared from the contagion (see Supplementary Figs. S4–S5).

### Economic impact of contagion and mobility restrictions before and during lockdown

Following results of the previous section, we first analyze how the epidemic directly impacts the Italian economy by decreasing the disposable labor force and, secondly, how mobility restriction measures have reshaped the Italian economy during the lockdown.

**Figure 3:**
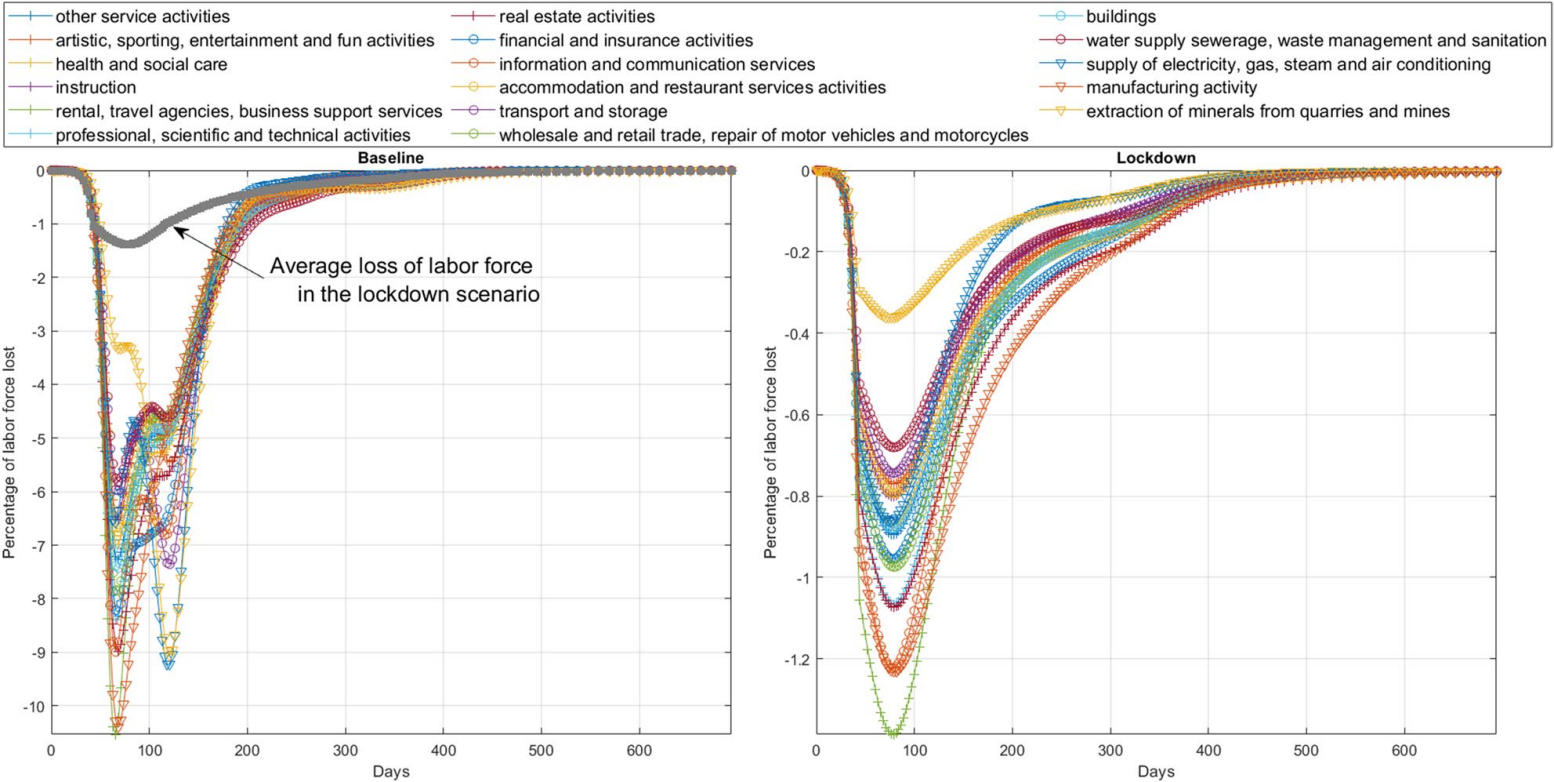
Labor force reduction due to infection evolution. The figure shows the simulated fraction of workers lost in each economic sector as they are infected. In particular the left panel reports the losses suffered in case of no lockdown measures while the right panel reports the percentage of workers lost in case mobility restriction measures are put in place. In the latter case we assume that the lockdown lifting fully restore the mobility flows while the transmission rate is still at the lockdown value. The number of infected people per district is reported in Supplementary Fig. S3.

As Eq. (1) indicates, only workers who are not infected by the virus could potentially produce. As a pictorial example, we report in Fig. 3 the simulated per sector percentage loss of labor force due to the epidemic depending on whether lockdown measures are applied or not, i.e. the relative difference for each sector $\nu$ among ${{\tilde{L}}}^{\nu } = \sum _{i}{\tilde{L}}_{i}^{\nu }$ measured with or without mobility restrictions. In both cases the manufacture sector and rental and travel activities are the most affected. We also notice that in absence of restrictive measures (left panel of the figure), a second peak of the infection would impact the extraction and energy supply sectors. Overall, results show that NPIs effectively mitigate the loss of labor force down to − 1.5% compared to more than − 10% in a scenario with no restrictions (see also Supplementary Fig. S7 which reports workers lost in different regions and economic sectors).

**Figure 4:**
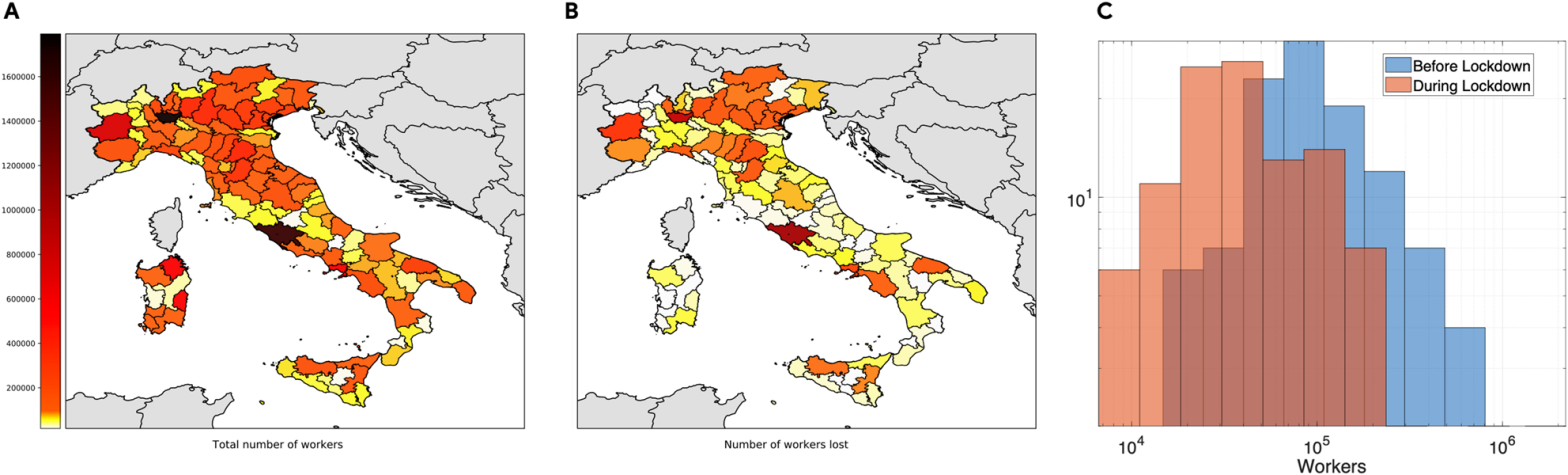
Labor force reduction due to mobility restriction policies. The figure represents the labor force reduction during the lockdown phase. Panel (**A**) shows the distribution of the total number of workers per each district, whereas panel (**B**) shows the distribution of the number of workers lost due to national lockdown. Darker colors correspond to higher values (in logarithmic scale). Finally panel (**C**) shows, in log–log scale, the workers’ distribution in the Italian districts in normal times (blue) and during (orange) the lockdown phase (when only essential sectors were allowed to produce).

However, as Eq. (2) suggests, the labor force is not only affected by the epidemic evolution, but it is also impacted by the policy measures put in place to circumvent the spread. Indeed, during the lockdown only a few sectors defined as essential by the Italian establishment^32,50^ were allowed to continue their activities (thus allowing workers to move). Figure 4 shows the labor force reduction due to lockdown policy measures. Panel A shows the distribution of the total number of workers per each district where darker colors correspond to higher values, whereas panel B the number of workers lost in the case of national lockdown. Panel C of Fig. 4 reports the distribution of working individuals in the Italian peninsula during “business as usual” times (i.e., pre-lockdown) and when lockdown applies (and only essential sectors are active). We can notice that lockdown measures strongly impact the labor force, but not in a homogeneous manner. Districts with an economy mainly grounded on the production of services suffer lower losses, while manufacturing oriented districts display a heavy reduction of the labor force, with the exception of Sardegna, a region which relies mostly on tourism.

The combined effect on the Italian labor force of the epidemic and mobility restriction policies per region is reported in Supplementary Fig. S6. In particular, the figure shows the workers’ reduction in absence (left) and presence (right) of lockdown measures. From the figure it clearly appears that national lockdown strongly impacts on the actual labor force, doubling the loss of workers w.r.t a scenario where the epidemic only might reduce the number of available workers. Thus, the net outcome of applying mobility restrictions is to considerably decrease the number of actual workers.

The two contrasting effects of the epidemic and lockdown measures on the economy are translated into monetary losses by multiplying the per-capita daily income (at the district level) and the actual labor force in the district $W_{i}(t)=w_{i} \cdot L_{i}(t)$. The geographical dispersion of the income on the Italian territory is shown in Supplementary Fig. S8. Notice that northern districts have the highest income, especially those located in Lombardia which, however, is also the area most affected by the virus.

**Figure 5:**
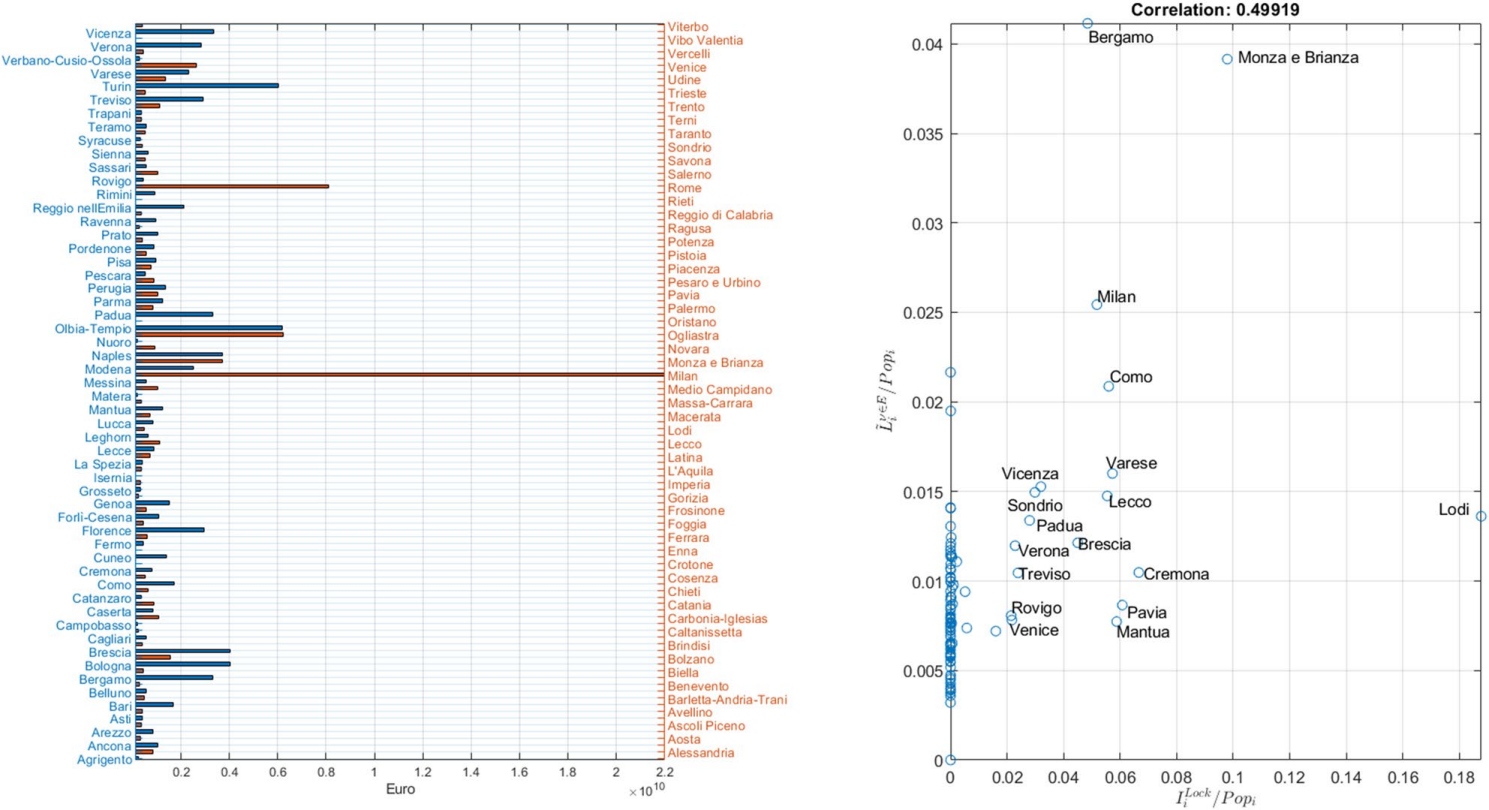
Economic losses per-district due to the epidemic and mobility restrictions. The figure, in the left panel, reports the aggregate economic losses (in euro) per district, focusing on the *Pre* and *Lock*down phases only. The right panel shows the scatter plot between the fraction of workers in essential sectors and the fraction of infected per district.

Figure 5 reports, in the left panel, the economic losses, derived from both the epidemic and mobility restrictions, and suffered by each district during the pre and lockdown phases. These losses incorporate both the epidemic effect on production and the hospitalization costs and are computed according to Eq. (4). Lombardy districts suffer the highest losses, being the most affected by the epidemic, followed by Rome which, on the contrary, mostly suffers due to mobility reductions. In the right panel of Fig. 5, we show the correlation of the fraction of workers belonging to essential sectors, and thus allowed to produce during the lockdown, and the number of infected individuals in each district. We observe that a few districts in the North-East have high numbers of both moving workers and infected individuals, thus indicating SARS-COV-2 as the primary source of economic losses, whereas in the rest of the peninsula the lack of correlation prompts to mobility restrictions as the main cause for economic backslashes.

### Scenario analysis for lockdown lifting

So far, we did not take into account the uncertainty related to Phase-2, in which information on transmission rates and mobility restrictions are not available in advance. Therefore, we evaluate the impact of lockdown lifting through a scenario analysis, i.e., we simulate the number of infected individuals after lifting the lockdown by varying parameters $l^{Unlock}$ and $\beta ^{Unlock}$ according with $\delta _{1}$ and $\delta _{2}$, i.e., such that $l^{Unlock}=\delta _{2}l^{Pre}$ and $\beta ^{Unlock}=\delta _{1}\beta ^{Pre}$ while fixing $\gamma =0.1$ and $r^{Unlock}=r^{Pre}$. We evaluate the model on a grid of 2500 points in the space $[0.01, 1]\times [0.5, 1]$ (taking 50 points linearly distributed on each axis), recording for each simulation the evolution of the number of infected inhabitants in each district. This step is instrumental for computing the fraction of available workers during Phase-2.

**Figure 6:**
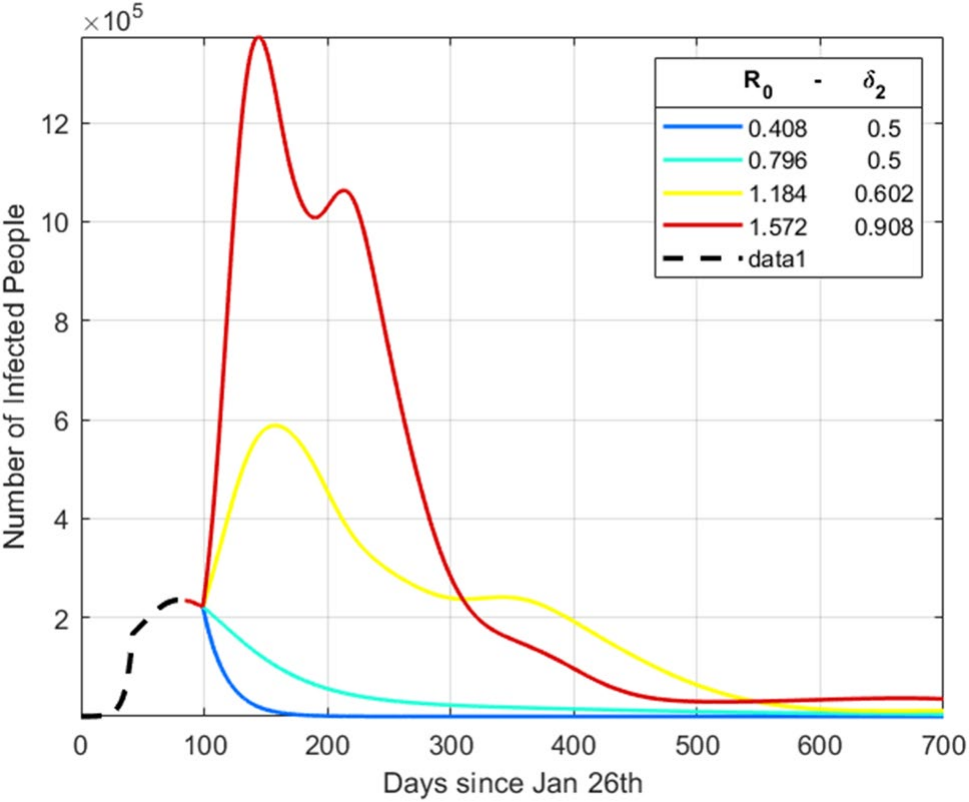
Evolution of the number of infected individuals in Italy for simulated Phase-2 scenarios. The figure shows the number of infected individuals for the entire peninsula when $\delta _{1}$ and $\delta _{2}$ vary, thus affecting both the mobility flows and the transmission rate. For sake of interpretability, instead of $\delta _{2}$ we report the related $R_{0}$ computed as $R_{0}=\delta _{2} \cdot \beta ^{Pre} / \gamma$.

Figure 6 shows simulation results for four representative combinations of $\delta _{1}$ and $\delta {2}$. For sake of interpretability, we mapped the parameter $\delta _{2}$ directly to the related $R_{0}$ since $R_{0}=\delta _{2} \cdot \beta ^{Pre} / \gamma$. As expected, values of $\delta _{2}$, which are associated with a $R_{0}$ lower then 1, produce a vanishing epidemic, while higher values of $\delta _{2}$ sustain a long lasting epidemic. Moreover, $\delta _{1}$, beside affecting the mobility rate, does not really impact on the number of infected individuals.

**Figure 7:**
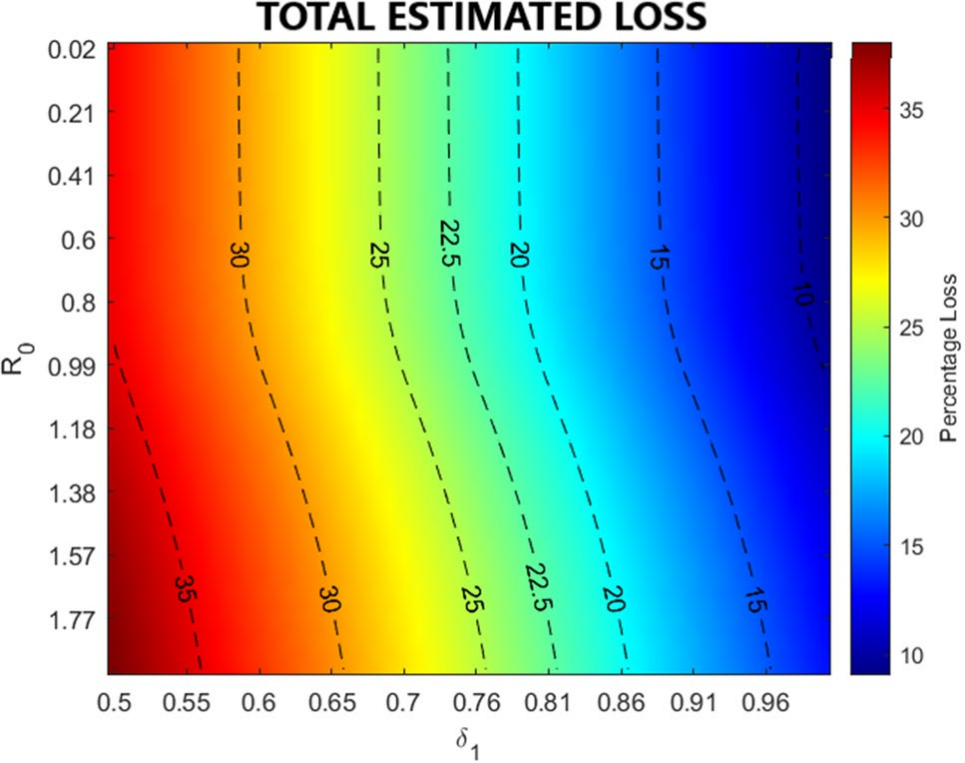
Simulation of economic losses for Italy in different Phase-2 scenarios. The figure shows the aggregate economic loss for Italy due to the SARS-COV-2 crisis as parameters $\delta _{1}$ and $\delta _{1}$ vary, accordingly affecting the mobility flows, the actual number of workers and the transmission rate. For sake of interpretability instead of $\delta _{2}$ we report the related $R_{0}$ computed as as $R_{0}=\delta _{2} \cdot \beta ^{Pre} / \gamma$. Contour lines are plotted in black dashes lines and define isolines of losses. The colorbar maps colors into percentage aggregate economic loss.

We further leverage our pool of simulations to model both the actual labor force and the economic loss in each district using Eqs. (2) and (4). In Fig. 7 hospitalization costs and income losses are combined in the simulations (see also Supplementary Figs. S9–S10) to provide the Italian aggregated economic loss for salary workers for different pairs of values of $\delta _{1}$ and $\delta _{2}$. The color scale describes the percentage of economic loss (ranging from red to blue as losses decrease) and contour lines define isolines of losses (i.e., couples of parameter values that induce the same aggregate economic loss for the country). The total economic loss is computed as $\sum _{i}\sum _{t}\Delta _{i}(t)$, where the parameter $\Delta _{i}(t)$ identifies the economic loss (the difference between the epidemic-free economic outcome and the actual outcome) suffered by district *i* at simulation time *t*. Again, for sake of interpretability, we mapped the parameter $\delta _{2}$ to the related $R_{0}$.

For increasing values of $\delta _{2}$ (and therefore of $R_{0}$), we observe in the simulations an increasing economic loss, since a higher number of infected individuals negatively affects the amount of available workers while increasing the impact of hospitalization costs. On the other hand, higher values of $\delta _{1}$ correspond to lower economic losses. Despite the fact that $\delta _{1}$ impacts on the mobility parameter ${\mathbf {l}}$ in the SIR model, thus facilitating contagion spread, it also influences the actual labor force in a positive way by allowing more people to reach workplaces. Overall, the net effect on the economy suggests that the economic loss decreases as long as $\delta _{1}$ increases.

**Figure 8:**
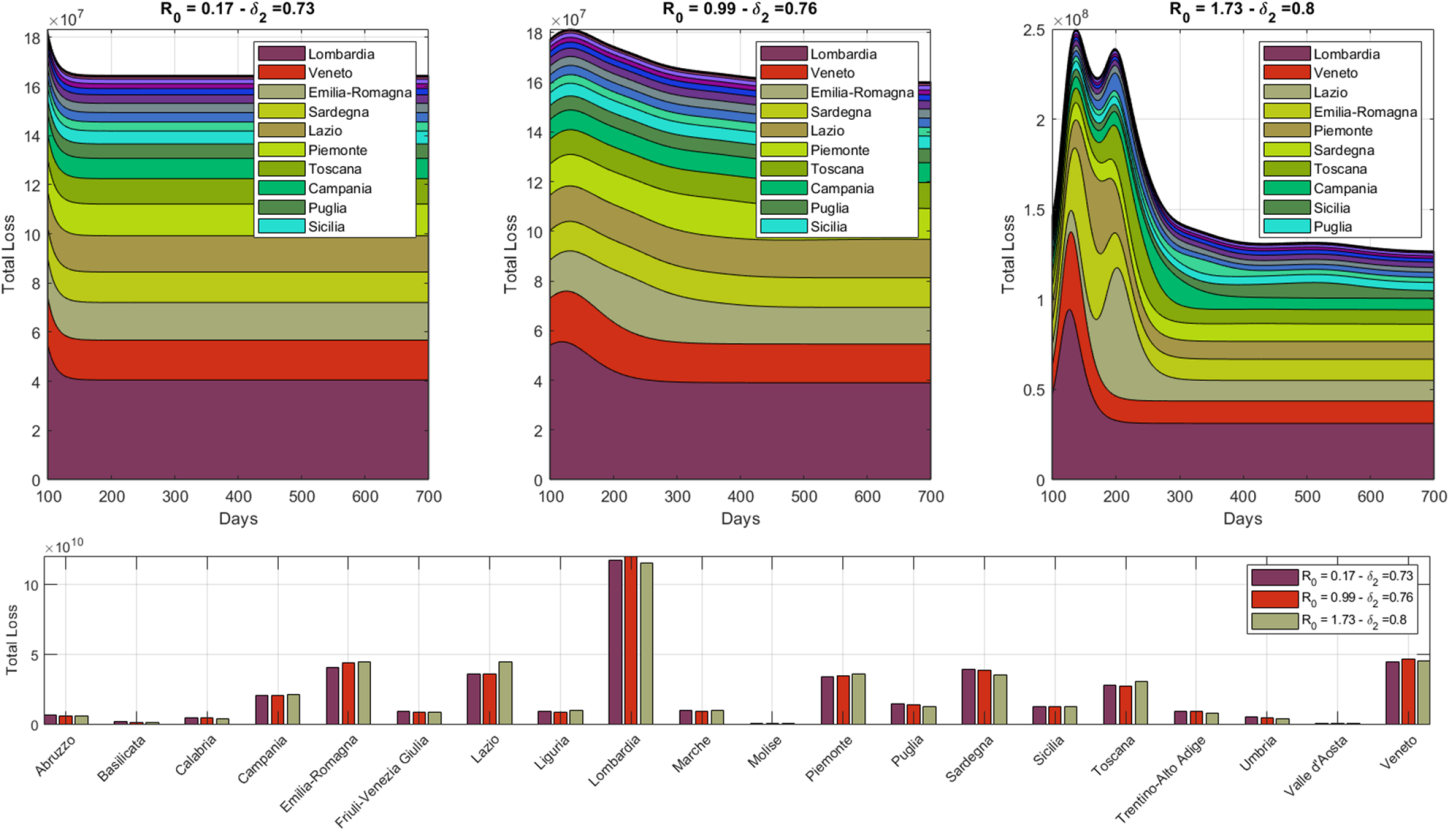
Simulation of economic losses for each region in different Phase-2 scenarios. The figure shows, in the upper panels, the dynamic of economic losses at the regional level for different combinations of parameters $\delta _{1}$ and $\delta _{2}$ (which generate the same total loss at the aggregate level). The lower panel reports regional losses aggregated over time for the three selected scenario cases. For sake of interpretability instead of $\delta _{2}$ we report the related $R_{0}$ found as $R_{0}=\delta _{2} \cdot \beta ^{Pre} / \gamma$.

Finally, we compare the evolution of the aggregate economic loss across regions in Phase-2 for different simulation scenarios keeping constant the total aggregated loss. Indeed, despite the fact that multiple configurations of $\delta _{1}$ and $\delta _{2}$ can produce a given aggregate loss for Italy, each couple of values determines a different geographical impact of the epidemic (see Supplementary Fig. S11) and thus of local economic backslashes. Figure 8 reports the simulated evolution of the economic loss in each region for three different configurations of $\delta _{1}$ and $\delta _{2}$ which produce the same aggregate economic reduction of about $22.5\%$ (see Fig. 7). Top panel shows the evolution of daily regional losses during three distinct Phase-2 scenarios, stacked in decreasing order according to their aggregated value at the end of the simulation $t={{\mathscr {T}}}$; total loss for each scenario is provided in the bottom panel. We observe that Lombardia and Veneto are the most severely hit in all scenarios, being the richest in terms of income and the most affected by the epidemic. When the epidemic is less contained (middle and right panels) we observe a strong impact, which is distributed among densely populated regions, that are located along the high-speed rail, from North (Piemonte) to Center (Toscana and Lazio) and South (Campania) of Italy.

## Discussion

The political debate on the transition to a post-lockdown phase (so called Phase-2) is affected by several unanswered questions on the timing and modality in which governments should lift mobility restrictions. On the one hand, a second wave of the epidemic could take place after lifting the restrictions. On the other hand, prolonged mobility restrictions would damage the economy. The uncertainty on the economic consequences of a second wave of restrictions is strengthened by the complexity of the global economy, where direct losses to a specific sector also entail second order effects to other sectors and to international trade partners. Including all these variables in a scenario analysis would require complex dynamic macroeconomic models in which policy alternatives embed welfare functions and citizens utility preferences, along with the impact of investments and fiscal interventions, heterogeneity of policies across regions, and macroeconomic unbalances from world trade shutdown.

Against this background, this paper proposes an illustrative micro-level framework to study the impact of possible alternative policy responses. We recognize that economic losses arise mainly from two main sources: the number of infected people and the restrictions in mobility that prevent individuals to work. Mobility restrictions, at a first glance, are beneficial for containing high losses in the number of available workers as they mitigate the epidemic spreading. However, restrictive policies have also a direct detrimental effect on economy as they force non-infected workers to vacate workplaces. Our study reveals that the aggregate reduction of disposable income can vary from − 10% up to − 40% in the worst case, and that the outcome is the result of non-linear interactions between mobility policies and infection transmission rates. In addition, we show that different combinations of parameters producing a given economic loss at the national level correspond to heterogeneous regional losses. These effects are shown to depend on the geographical impact of the disease and of the socio-economic structure of each territory. Despite the simple SIR model governing the spreading disease and the inherent approximation due to how we track mobility, our findings contribute to inform policy design as they suggest the adoption of geographically tailor-made policy actions instead of a one-rule-fits-all approach to mobility restrictions.

## Methods

### A SIR metapopulation model for Italian districts

The spatial structure of populations is a key ingredient for understanding the dynamical spreading of an epidemic, in particular in a territory where areas have a high degree of heterogeneity, such as the Italian peninsula^66^. Districts differ not only in their number of inhabitants, but also for what concerns transportation networks and commuting patterns among areas. To account for such heterogeneity we leverage a Susceptible-Infected-Recovered (SIR) model with metapopulation^13,14,16,19,33,67^ by subdividing the Italian population into districts. Each district has its own independent epidemiological dynamics, and commuters can further spread the disease into other districts. Since our main focus is on the economic effect of the SARS-COV-2 epidemic, we keep the transmission scheme as simple as possible to avoid confounding effects^68^.

Let $S_{ij}$, $I_{ij}$ and $N_{ij}$ be the number of susceptible, infected, and total hosts currently in district *i* that live in district *j*. The metapopulation SIR model is described by the following equations:

5 $$\begin{aligned} \begin{aligned} \frac{dS_{ii}}{dt}&=-\beta _{i}S_{ii}\frac{{\sum }_{j}I_{ij}}{{\sum }_{j}N_{ij}} -{\sum }_{j}l_{ji}S_{ii}+{\sum }_{j}r_{ji}S_{ji}\\ \frac{dS_{ij}}{dt}&=-\beta _{i}S_{ij}\frac{{\sum }_{j}I_{ij}}{{\sum }_{j}N_{ij}} +l_{ij}S_{jj}-r_{ij}S_{ij}\\ \frac{dI_{ii}}{dt}&=-\beta _{i}S_{ii}\frac{{\sum }_{j}I_{ij}}{{\sum }_{j}N_{ij}} -\gamma I_{ii}-{\sum }_{j}l_{ji}I_{ii}+{\sum }_{j}r_{ji}I_{ji}\\ \frac{dI_{ij}}{dt}&=-\beta _{i}S_{ij}\frac{{\sum }_{j}I_{ij}}{{\sum }_{j}N_{ij}} \gamma I_{ij} +l_{ij}I_{jj}-r_{ij}I_{ij}\\ \frac{dN_{ii}}{dt}&= - {\sum }_{j}l_{ji}N_{ii}+{\sum }_{j}r_{ji}N_{ji}\\ \frac{dN_{ij}}{dt}&= l_{ij}N_{jj}-r_{ij}N_{ij} \end{aligned} \end{aligned}$$

Therefore, the number of recovered individuals in district *i* that live in district *j* is $R_{ij}=N_{ij}-I_{ij}-S_{ij}$.

Matrices ${\mathbf {l}}$ and ${\mathbf {r}}$ determine the rate at which individuals leave from and return to their home district, respectively. Specifically, ${\mathbf {l}}$ includes all types of workers, both smart workers and not. Also, each entry $\beta _{i}$ in vector $\varvec{\beta }$ represents the transmission rate for the *i*th district. Finally, the parameter $\gamma$ is the recovery rate and it is assumed to be district independent.

### Human mobility and parameters calibration

Real data is of paramount importance to calibrate the model^69^, i.e., to ensure that the output is realistically informative on the epidemic spreading. Thus, we leverage a massive dataset of near real-time observations provided by Facebook^24^ through its Data for Good program. In this way we can tune model parameters concerning the mobility rates before and during the lockdown phase, ${\mathbf {l}}^{Pre}$, ${\mathbf {r}}^{Pre}$ and ${\mathbf {l}}^{Lock}$, ${\mathbf {r}}^{Lock}$, respectively.

Facebook Disease Prevention maps provide information on movements of individuals with their geo-positioning option enabled during the period of observation, which goes from February 24th to March 23rd. Our dataset consists of approximately 800,000 distinct observations (recorded with a 8-h frequency) covering movements of nearly 4 million daily individuals (on average) across 3000 Italian municipalities. This data is not publicly available but it can be released to non-profit organizations and academics upon agreement with Facebook.

Each observation in the Facebook dataset describes movements of individuals across tiles^70^, which we aggregated at increasing territorial levels to perform our analyses. The aggregation process consists in summing together all observations belonging to the same territorial level (e.g. municipalities, districts), hence it does not entail any loss of information as each tile is assigned to a unique territorial unit. Furthermore, we represent mobility using a directed weighted graph formulation where nodes are territorial units and edges are weighted based on the amount of traffic of individuals flowing between two locations.

For the mobility analysis we use data at the municipality level, whereas in order to simulate our epidemic model we further aggregate mobility flows among municipalities at the district level. In particular, we built two snapshots corresponding to *Pre* and *Lock* phases by aggregating together daily mobility over two 14-day windows, before and after the day of the intervention (March 9th), respectively. Let $\Gamma _{xy}^{t}$ be the index of mobility from municipality *x* to municipality *y* at day *t*, i.e. the number of individuals moving between the two locations. The estimates of entries in matrices ${\mathbf {l}}^{Pre,Lock}$ are then obtained as the normalized average of the out-of-diagonal flows across cities after mapping each municipality to the corresponding district. In formulae:

6 $$\begin{aligned} {\tilde{l}}^{T}_{ij}= & {} \sum _{x \in i}\sum _{y \in j}\sum _{t \in T}\frac{\Gamma _{xy}^{t}}{T} \end{aligned}$$

7 $$\begin{aligned} l^{T}_{ij}= & {} \frac{{\tilde{l}}^{T}_{ij}}{\sum _{i}\sum _{j}{\tilde{l}}^{T}_{ij}}, \end{aligned}$$

where $T=\{Pre,Lock\}$. Hence ${\tilde{l}}^{T}_{ij}$ represents the probability of an individual to leave district *i* at time *t* for district *j*.

The binary version of the matrix ${\mathbf {l}}^{T}$ instead has been used to calibrate ${\mathbf {r}}^{T}$, under the hypothesis that people return entirely to their home district at the end of the day:

8 $$\begin{aligned} r^{T}_{ij} = {\left\{ \begin{array}{ll} 1 &{} \text{ if } l^{T}_{ij}>0 \\ 0 &{} \text{ otherwise }. \end{array}\right. } \end{aligned}$$

The parameter $\gamma =\tau ^{-1}_{I}$ is set equal to 0.1 assuming an infection time ($\tau _{I}$) duration of 10 days both for the pre-lock and lockdown periods. This is in accordance with the range of infection durations reported by the European Centre for Disease controls, which is between 5 and 14 days^26^.

For calibrating the district specific transmission rate during the pre-lockdown phase ($\beta _{i}^{Pre}$), we applied regional reproduction numbers from Ref.^27^ $R_{0}^{z}$, where *z* indicates an Italian region, for each district located in region *z*. In formula:

9 $$\begin{aligned} \beta _{i}^{Pre}= {\left\{ \begin{array}{ll} R_{0}^{z} \cdot \gamma&\text{ if } i \in z. \end{array}\right. } \end{aligned}$$

For calibrating the transmission rate during the lockdown phase $\beta _{i}^{Lock}$, we assumed a reduction of $\beta _{i}^{Pre}$ proportional to the decrease of the internal mobility in each district (i.e., the amount of traffic flowing between distinct municipalities of the same district), thus $\beta _{i}^{Lock} = \alpha _{i}\beta _{i}^{Pre}$ where:

10 $$\begin{aligned} \alpha _{i} = 1 -\frac{l_{ii}^{Pre}-l_{ii}^{Lock}}{l_{ii}^{Pre}}. \end{aligned}$$

Despite these strong assumptions, our proposal is in line with Ref.^29,69^, where authors assume that the effect of national lockdown and other governmental interventions, such as school closure, social distancing or self-isolation, is to reduce the original $R_0$ by a proportional factor (in Supplementary Fig. S3 we report the calibrated values of $R_{0}$ at regional level for the pre-lockdown and lockdown phases). Moreover, this approximation ensures that the average transmission rate during the lockdown phase is $\approx \;0.78$, which is compatible with a vanishing epidemic that would allow to lift mobility restriction policies and start a post-lockdown phase^36^. Besides, the reduction of internal mobility estimated with Facebook data is in accordance with latest reports on the decreasing trend of Italian mobility provided by Google^71^. However, we remark that the model only aims to show the economic effect of different scenarios rather than providing an accurate forecasting of the epidemic evolution.

To study the economic impact of the epidemic after the lockdown phase, we perform simulations with values in-between two extreme cases. An over-pessimistic scenario in which mobility restrictions continue while the transmission rates turn back to their pre-lockdown values and an over-optimistic scenario in which mobility is fully restored to pre-lockdown values while transmission rates are kept to the minimum reached during lockdown phase. Thus, let ${\mathbf {l}}^{Unlock}=\delta _{1} {\mathbf {l}}^{Pre}$, $\varvec{\beta }^{Unlock}=\delta _{2}\varvec{\beta }^{Pre}$ and ${\mathbf {r}}^{Unlock}={\mathbf {r}}^{Pre}$ be the parameters’ values during Phase-2. By varying $\delta _{1}$ and $\delta _{2}$ in the ranges [0.5, 1] and [0.01, 1], respectively, we obtain economic losses for different parameters’ combinations. We measure the economic impact of mobility restrictions mainly through labor force loss. This allow us to use actual data on the composition of the productive structure of the economy without relying on other assumptions on the behaviour of consumers and firms, given that these kind of measurements are still not available for Italy. By doing so, our results represent a lower bound for economic losses, given that they do not account for second order effects on aggregate demand due to the decrease in consumption caused by fall in wages and decrease in firms consumption of intermediate goods caused by fall in demand from consumers. On the other hand we do not consider two factors which may reduce the economic impact of restrictions: government interventions and the increasing availability of remote work. For both issues there was no data available and hence we have ignored the effect of government interventions and assumed that the fraction of remote work in each sector was the same.

## Supplementary information

Supplementary Information.

## Data Availability

Raw observations of human mobility are not publicly available, as they were provided by Facebook with an academic agreement through its “Data for Good” program (https://dataforgood.fb.com), whereas economic variables are publicly available on the ISTAT website (https://www.istat.it). Nevertheless, we can provide, upon request to the corresponding author, the origin-destination matrices which describe inter/intra-district mobility before and during lockdown, as well the entire set of economic variables and the code to reproduce our experiments and/or carry out further analyses.
