# Supplementary material for: After the lockdown: simulating mobility, public health and economic recovery scenarios
Source: Sci Rep. 2020 Oct 12;10:16950. doi: 10.1038/s41598-020-73949-6 (PMC7550600; doi:10.1038/s41598-020-73949-6)
Supplement: Supplementary file 1 — Supplementary Information. [file 41598_2020_73949_MOESM1_ESM.pdf]

Supplementary information

After the lockdown: simulating mobility, public health and economic recovery scenarios

Alessandro Spelta[1,2*], Andrea Flori[2], Francesco Pierri[2,3*], Giovanni Bonaccorsi[2], and Fabio Pammolli[2,4]

[1]Department of Economics and Management, University of Pavia, Via San Felice 7, 27100, Pavia, Italy
[2]Impact, Department of Management, Economics and Industrial Engineering, Politecnico di Milano, Via Lambruschini, 4/B, 20156, Milan, Italy
[3]Department of Electronics, Information and Bioengineering, Politecnico di Milano, Via Giuseppe Ponzio 34/5, 20133, Milan, Italy
[4]CADS, Joint Center for Analysis, Decisions and Society, Human Technopole, Via Cristina Belgioioso, 171, 20157, Milan, Italy
*Corresponding author: alessandro.spelta@unipv.it, francesco.pierri@polimi.it

1 Supplementary Figures

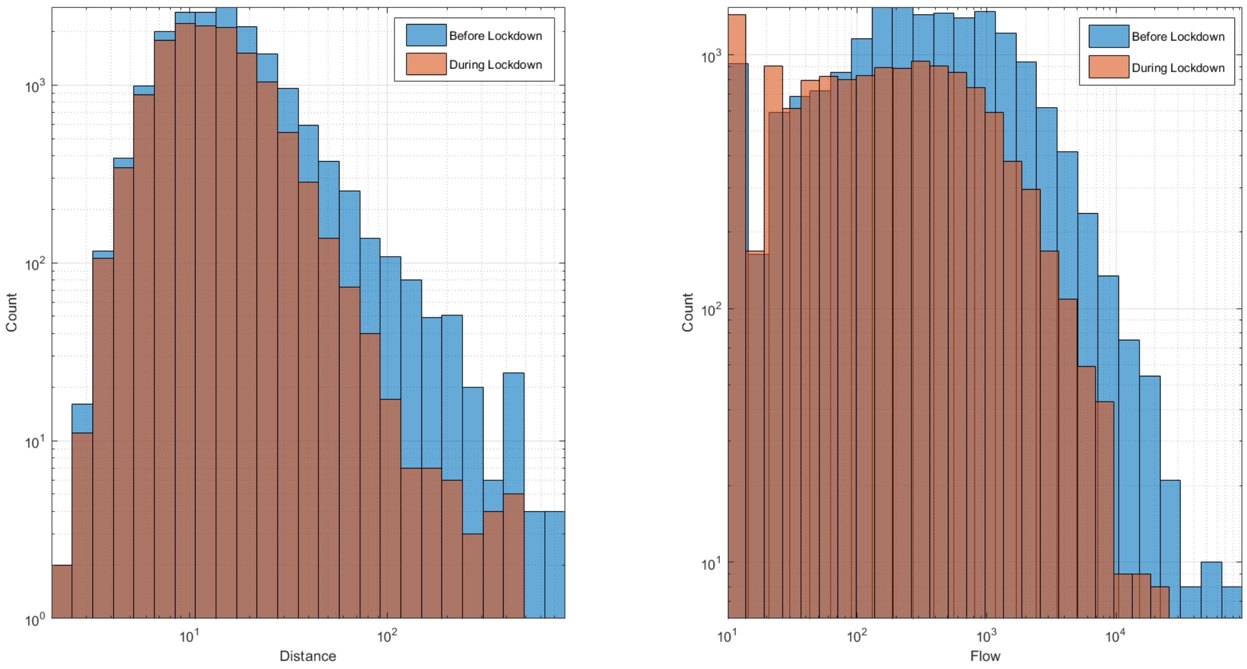

Supplementary Figure 1: **Distributions of movements in Italy before and during the lockdown.** The figure shows the reduction in national mobility induced by the lockdown measures of March 9th. The right panel shows the distributions of the travelled distance (in Km) and left panel reports the number of movements before the intervention (blue) and during the lockdown phase (orange).

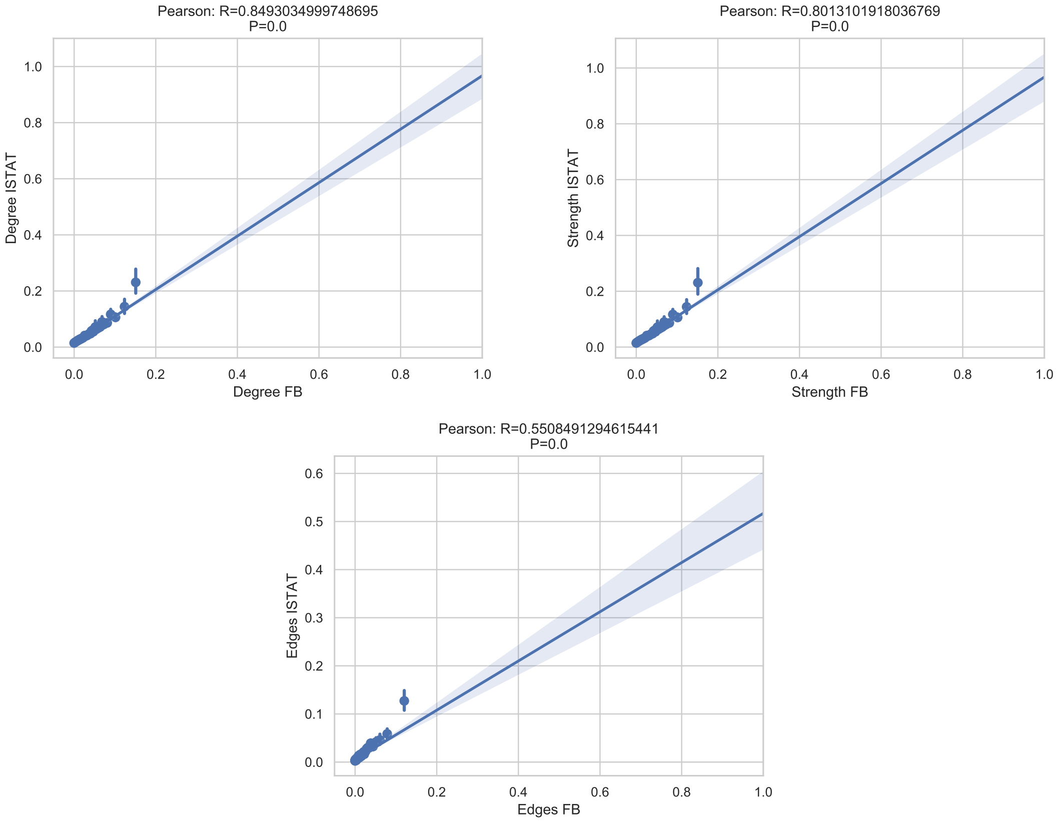

Supplementary Figure 2: Scatterplot of Degree and Strength of nodes, and Weight of edges in Facebook mobility network before lockdown (x-axis) vs ISTAT commuting network (y-axis). Pearson's correlation coefficient and pvalue are shown in the heading.

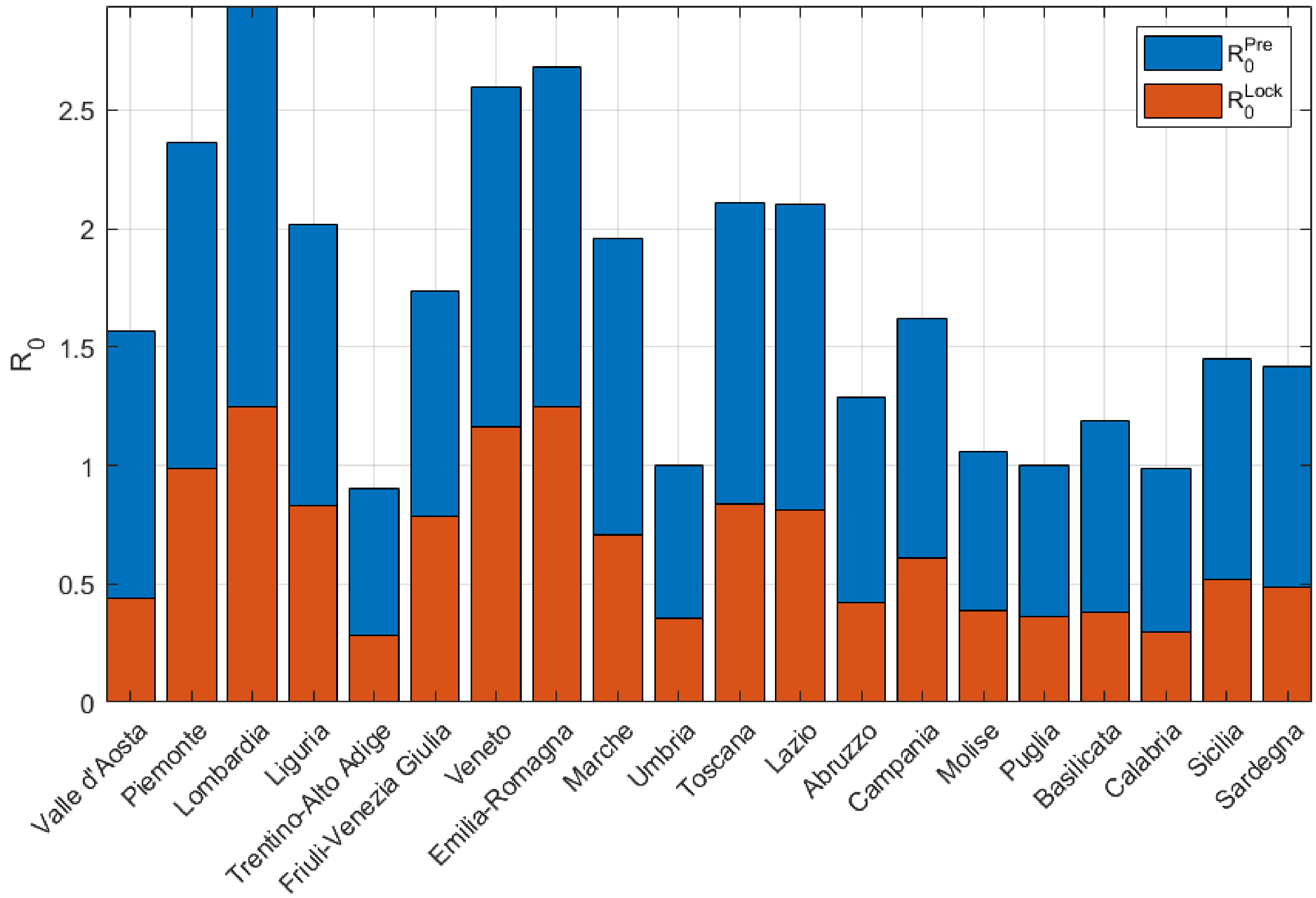

Supplementary Figure 3: **Calibrated $\mathbf{R_0}$.** The figure shows the value of the calibrated R_0 for the initial epidemiological phase R_0^{Pre} and for the lockdown phase R_0^{Lock}.

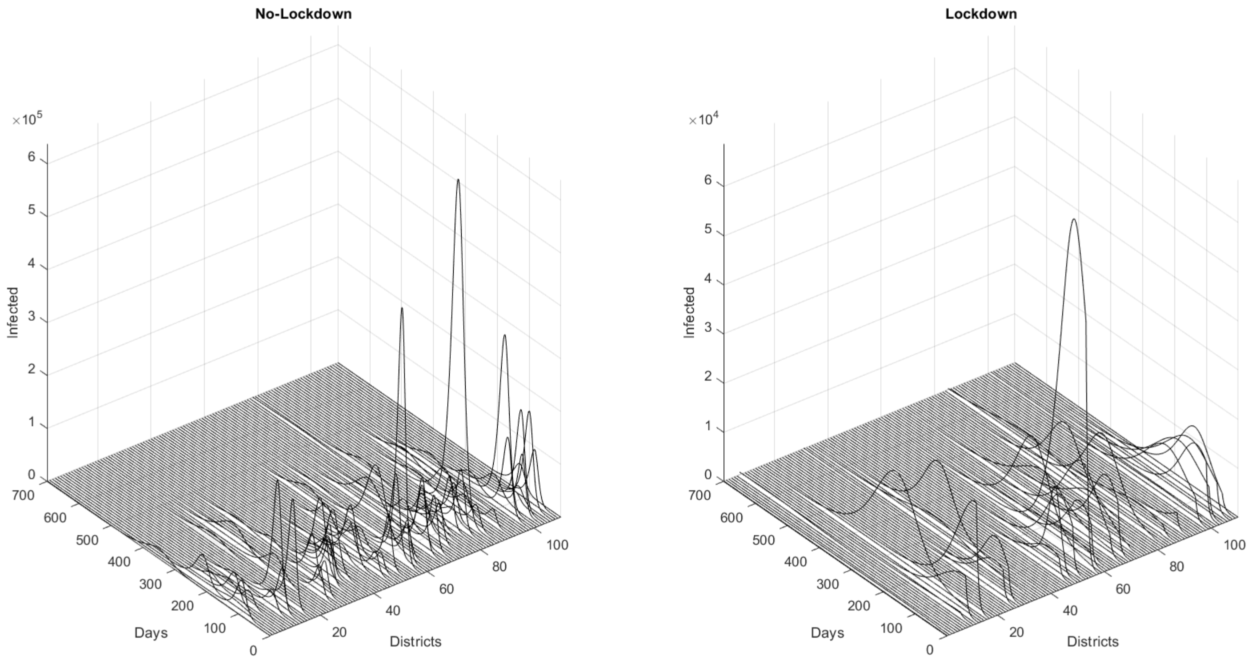

Supplementary Figure 4: **Temporal evolution of the epidemic in Italy** The figure shows the temporal evolution of the SARS-COV-2 in Italy in absence of lockdown measures (left panel) or in the case when mobility restriction policies are applied (right panel).

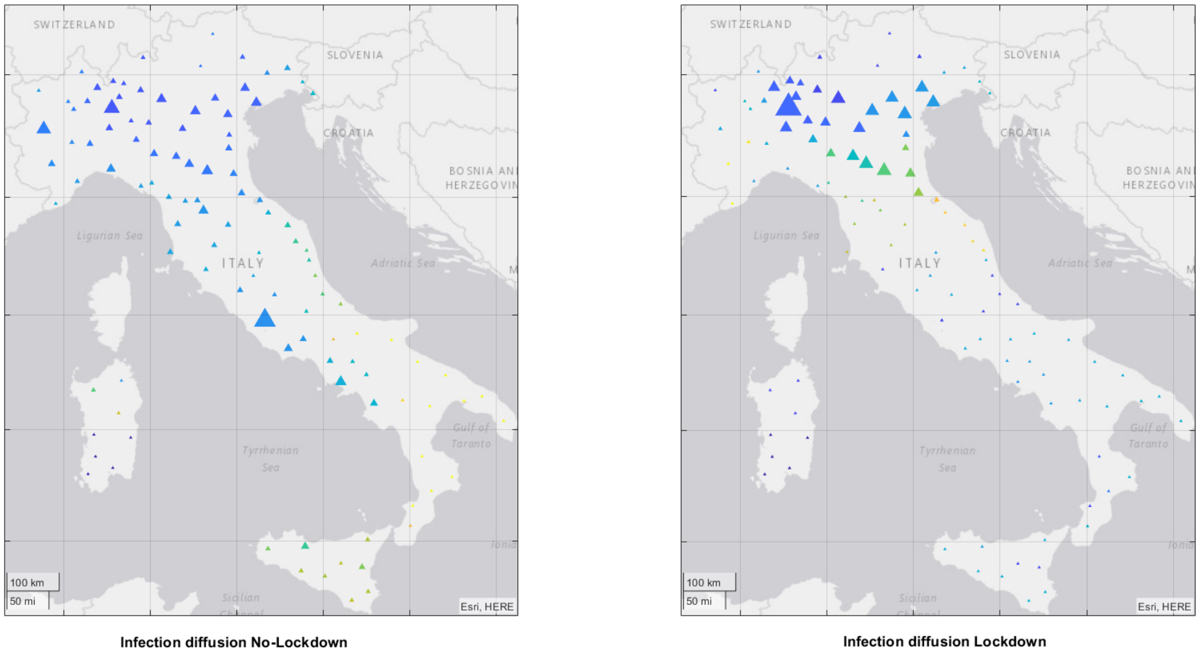

Supplementary Figure 5: **Spatial evolution of the epidemic in Italy.** The figure shows the spatial evolution of the SARS-COV-2 in Italy in absence of lockdown measures (left panel) or in case of mobility restriction policies (right panel). The triangles on the map geo-localize a province. The size of each triangle is proportional to the total number of infected individuals in the district and the color indicate the temporal occurrence of the peak (with darker colors corresponding to earlier periods). Plots were obtained using *geoplot* library of MATLAB (https://it.mathworks.com/help/matlab/ref/geoplot.html).

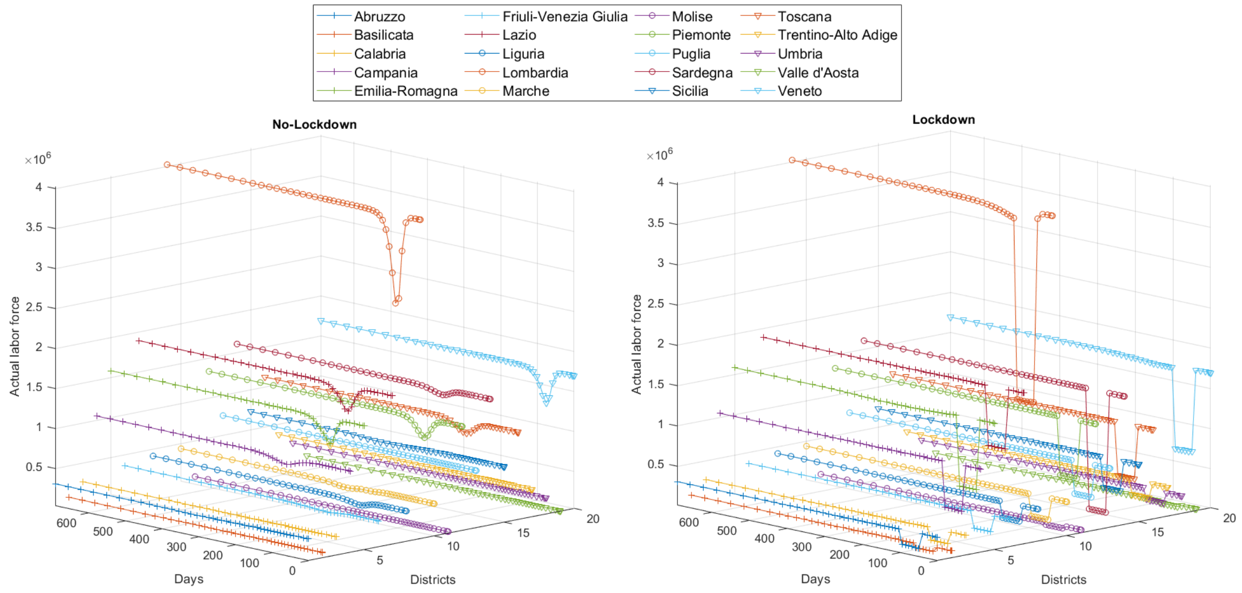

Supplementary Figure 6: **Labor force reduction per region due to both epidemic and mobility restriction policies.** The figure represents the dynamics of labor force in each region in two extreme cases. In the left panel we report workers' reduction in absence of lockdown measures. The right panel shows the number of available workers in case lockdown measures are put in place and then lifted, consequently restoring mobility flows without increasing the transmission rate above the lockdown value.

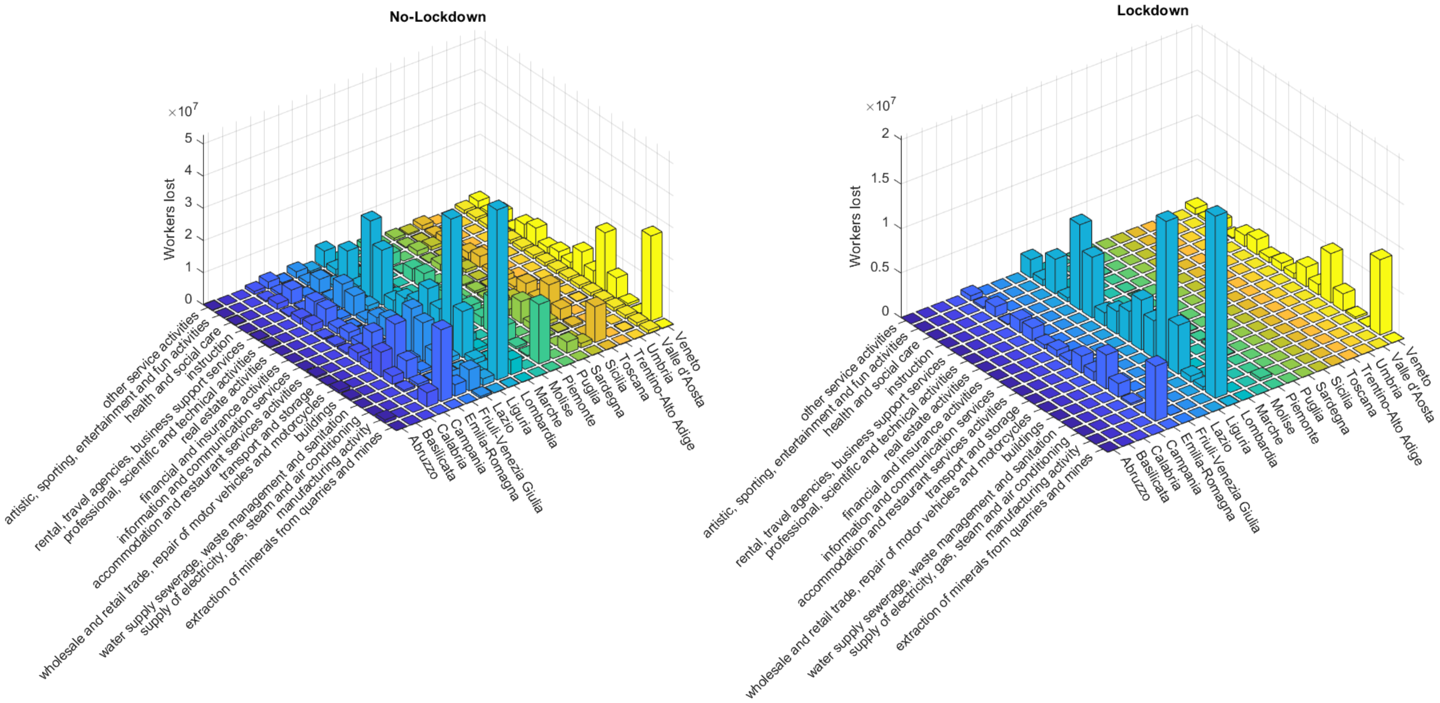

Supplementary Figure 7: **Workers lost per region and economic sector.** The figure shows the number of workers lost due to the SARS-COV-2 crisis in each region and economic sector in the two basic scenarios. In the left panel we report the workers lost in the case of absence of lockdown measures. The right panel shows the loss in the case of lockdown measures and with lockdown lifting which restores the mobility flows without increasing the transmission rate above the lockdown value.

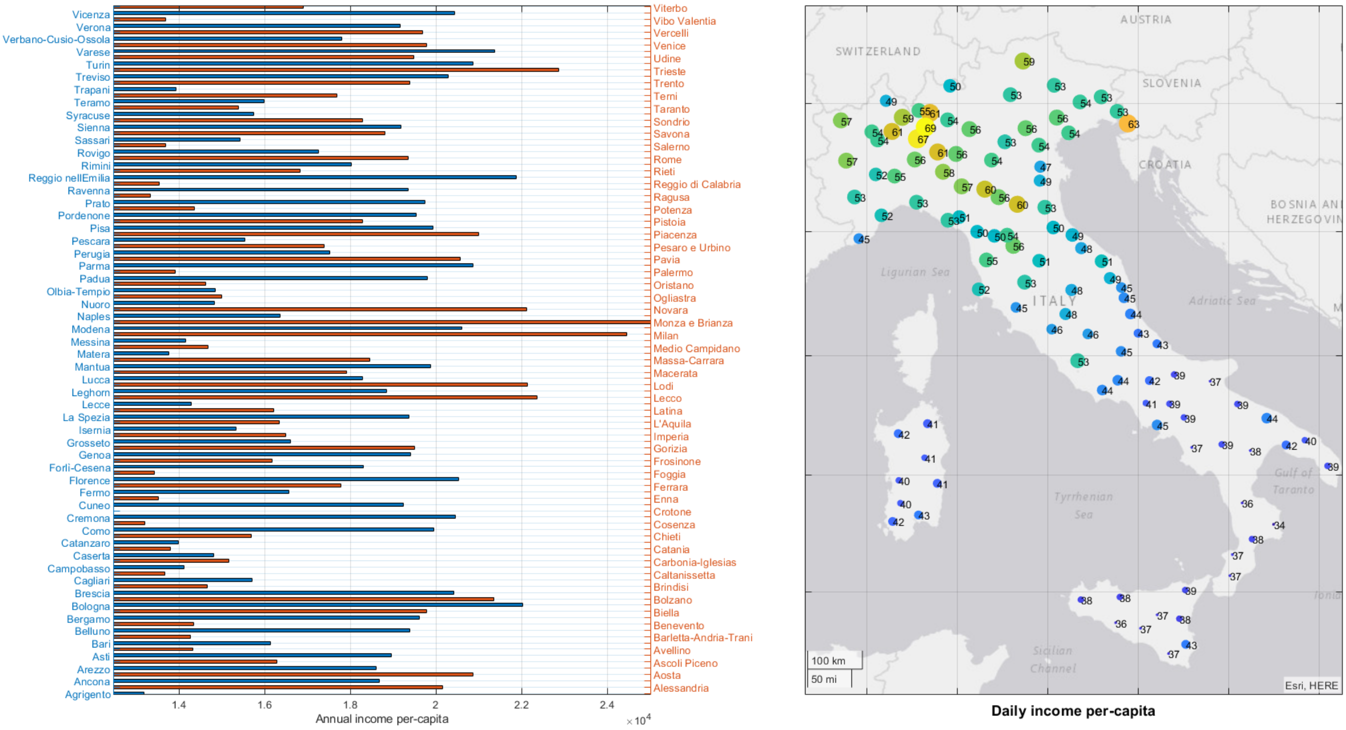

Supplementary Figure 8: **Average annual and daily income per district.** The figure shows the annual average income per district on the left and the daily average income on the right. In particular, the size of circles in the right panel is proportional to the daily income in each district. Right plot was obtained using *geoplot* library of MATLAB (https://it.mathworks.com/help/matlab/ref/geoplot.html).

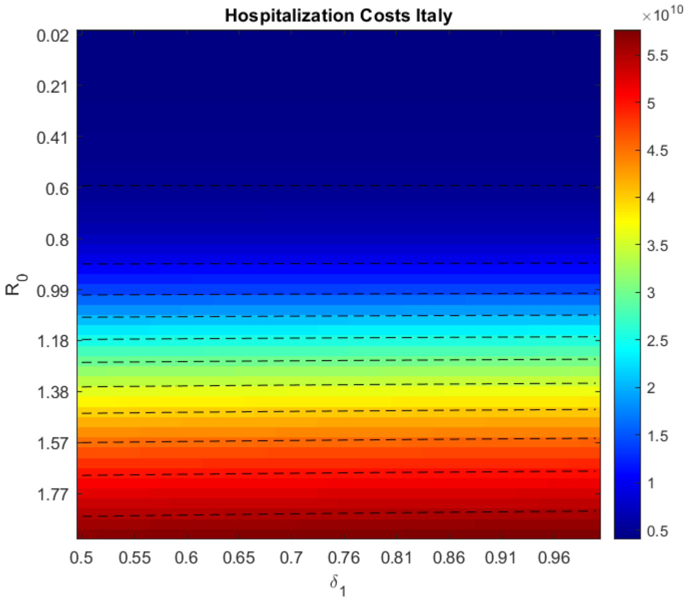

Supplementary Figure 9: **Hospitalization costs for Italy in different Phase-2 scenarios.** The figure shows the aggregate hospitalization costs for Italy due to the SARS-COV-2 crisis as long as parameters δ_1 and δ_2 vary, affecting the mobility flows, the actual number of workers and the transmission rate. For sake of interpretability instead of δ_2 we report the related R_0 found as $R_0 = \delta_2 \cdot \beta^{Pre}/\gamma$. Countour lines are plotted in black dashes lines and define isolines of losses. The colorbar maps colors into euro.

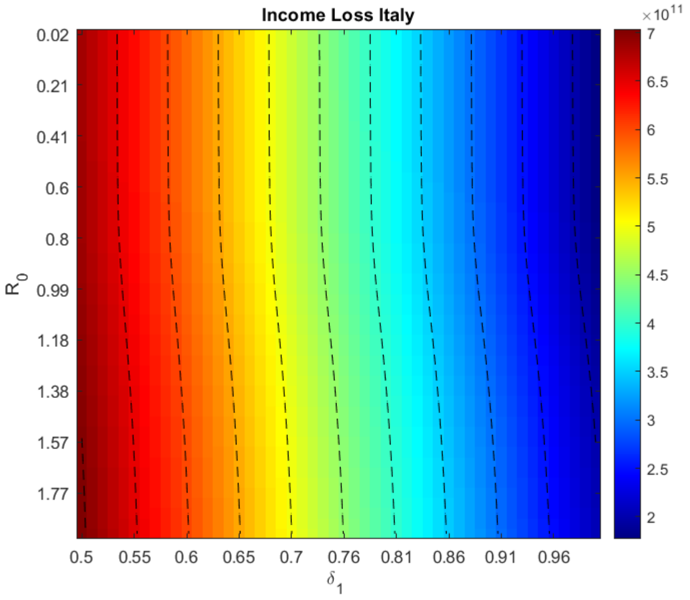

Supplementary Figure 10: **Income loss for Italy in different Phase-2 scenarios.** The figure shows the total income loss due to the SARS-COV-2 crisis as long as parameters δ_1 and δ_2 vary, affecting the mobility flows, the actual number of workers and the transmission rate. For sake of interpretability instead of δ_2 we report the related R_0 found as $R_0 = \delta_2 \cdot \beta^{Pre}/\gamma$. Countour lines are plotted in black dashes lines and define isolines of losses. The colorbar maps colors into euro losses.

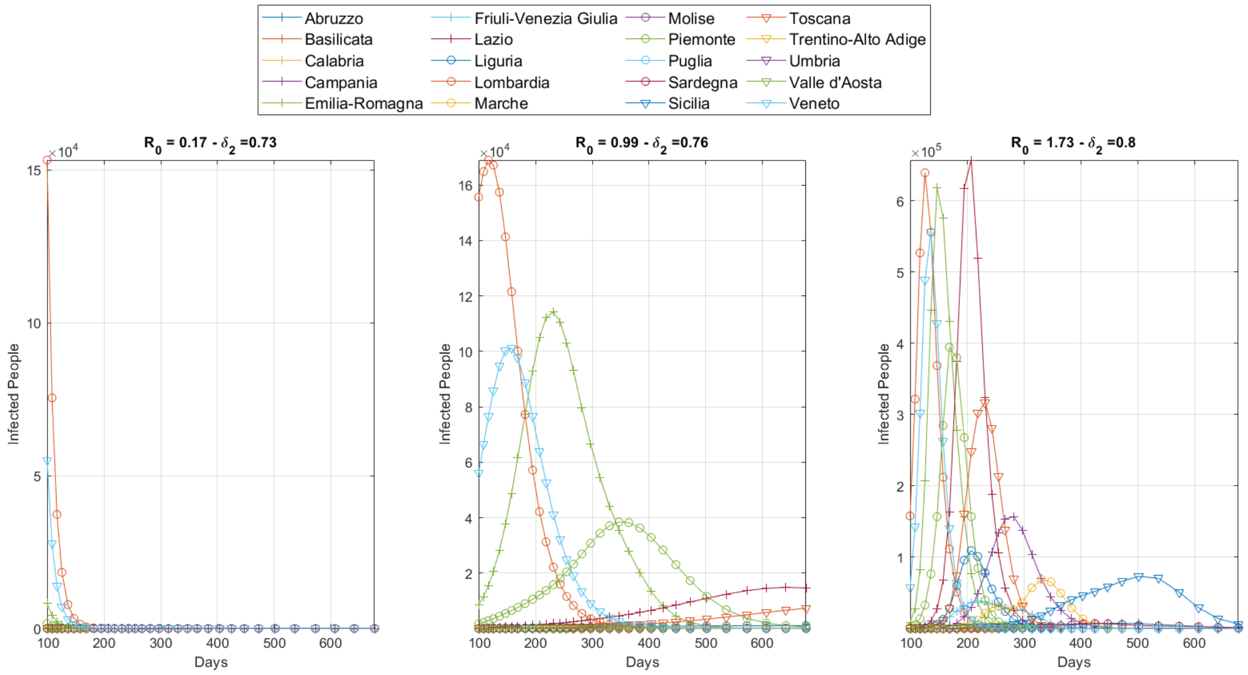

Supplementary Figure 11: **Regional infection dynamic in different Phase-2 scenarios.** The figure shows the regional infection dynamic for different combinations of parameters δ_1 and δ_2. For sake of interpretability instead of δ_2 we report the related R_0 found as $R_0 = \delta_2 \cdot \beta^{Pre}/\gamma$.
